# Improving User Experience of Virtual Health Assistants: Scoping Review

**DOI:** 10.2196/31737

**Published:** 2021-12-21

**Authors:** Rachel G Curtis, Bethany Bartel, Ty Ferguson, Henry T Blake, Celine Northcott, Rosa Virgara, Carol A Maher

**Affiliations:** 1 UniSA Allied Health and Human Performance Alliance for Research in Exercise, Nutrition and Activity University of South Australia Adelaide Australia

**Keywords:** virtual assistant, conversational agent, chatbot, eHealth, digital health, design, user experience, mobile phone

## Abstract

**Background:**

Virtual assistants can be used to deliver innovative health programs that provide appealing, personalized, and convenient health advice and support at scale and low cost. Design characteristics that influence the look and feel of the virtual assistant, such as visual appearance or language features, may significantly influence users’ experience and engagement with the assistant.

**Objective:**

This scoping review aims to provide an overview of the experimental research examining how design characteristics of virtual health assistants affect user experience, summarize research findings of experimental research examining how design characteristics of virtual health assistants affect user experience, and provide recommendations for the design of virtual health assistants if sufficient evidence exists.

**Methods:**

We searched 5 electronic databases (Web of Science, MEDLINE, Embase, PsycINFO, and ACM Digital Library) to identify the studies that used an experimental design to compare the effects of design characteristics between 2 or more versions of an interactive virtual health assistant on user experience among adults. Data were synthesized descriptively. Health domains, design characteristics, and outcomes were categorized, and descriptive statistics were used to summarize the body of research. Results for each study were categorized as positive, negative, or no effect, and a matrix of the design characteristics and outcome categories was constructed to summarize the findings.

**Results:**

The database searches identified 6879 articles after the removal of duplicates. We included 48 articles representing 45 unique studies in the review. The most common health domains were mental health and physical activity. Studies most commonly examined design characteristics in the categories of visual design or conversational style and relational behavior and assessed outcomes in the categories of personality, satisfaction, relationship, or use intention. Over half of the design characteristics were examined by only 1 study. Results suggest that empathy and relational behavior and self-disclosure are related to more positive user experience. Results also suggest that if a human-like avatar is used, realistic rendering and medical attire may potentially be related to more positive user experience; however, more research is needed to confirm this.

**Conclusions:**

There is a growing body of scientific evidence examining the impact of virtual health assistants’ design characteristics on user experience. Taken together, data suggest that the look and feel of a virtual health assistant does affect user experience. Virtual health assistants that show empathy, display nonverbal relational behaviors, and disclose personal information about themselves achieve better user experience. At present, the evidence base is broad, and the studies are typically small in scale and highly heterogeneous. Further research, particularly using longitudinal research designs with repeated user interactions, is needed to inform the optimal design of virtual health assistants.

## Introduction

### Background

Advancements in machine learning and artificial intelligence offer promise for delivering automated, tailored, convenient health assistance with an unprecedented level of sophistication and personalization and are already contributing to the transformation of health care [1]. Virtual assistants can be broadly defined as digital services designed to simulate human conversation and provide personalized responses based on input from the user. They can be programmed with structured conversations or to answer the user’s questions. Capabilities range from simple menu or multiple choice–based assistants to more sophisticated virtual assistants with natural language processing that recognize free speech or text. At present, virtual assistants are widely deployed in web-based banking and service settings, reducing reliance on staff by being available to answer consumers’ questions about products and services *on demand*. Virtual assistants are also increasingly being designed for various health applications, such as delivering cognitive behavior therapy for depression and anxiety [2], improving diet and physical activity [3], and conducting remote patient monitoring [4]. Despite the exciting potential for using virtual assistants for health purposes, the use of virtual assistants in health could be ineffective or even have unintended negative consequences if the technology does not meet the user’s needs and preferences.

The user experience of a virtual health assistant can be defined as the user’s perceptions and responses (eg, emotions, beliefs, preferences, and behaviors) that result from its use or anticipated use [5]. User experience is influenced by a range of factors, including presentation, functionality, and interactive behavior [5]. It is important to optimize the design of virtual assistants to provide a positive user experience and promote engagement. A growing body of evidence suggests that design characteristics that influence the *look and feel* of the virtual assistant, such as visual appearance, communication method, and language features, are an important consideration for design, as such design characteristics can significantly influence users’ psychological and emotional responses and engagement with technology-based applications [6,7]. In addition, although some design decisions may not affect the cost (eg, whether an avatar should be male or female), other decisions may have a major impact on the cost of designing a virtual health assistant (eg, whether an avatar should be animated with facial expressions). Understanding how such design characteristics influence user experience will assist in using finite health software development budgets most effectively.

Previous literature has proposed general guidelines for designing voice user interfaces [8] and accessible conversational user interfaces for different disability groups [9], as well as virtual assistants for specific purposes such as teaching [10] and in-vehicle assistance [11]. Optimal design techniques are likely to depend on the purpose of the virtual assistant [12,13]; therefore, recommendations specifically in the context of health are needed. Although research has examined methods of assessing the usability of virtual assistants in the health domain [14], clear guidelines on maximizing the user experience of virtual health assistants are lacking.

An important first step toward constructing guidelines for the development of virtual health assistants was achieved by the literature review conducted by ter Stal et al [15] in 2018, which aimed to identify the researched design characteristics for embodied conversational agents (virtual assistants that have an animated avatar) in health. The review provided a comprehensive overview of the existing literature, with results suggesting that speech and/or textual output and facial and gaze expressions were the most commonly researched design characteristics. The secondary aims of ter Stal et al [15] were to identify the outcome variables used in the research and the effects of the design characteristics. The authors concluded that, based on the immature body of evidence at the time, there was no consensus on the optimal design characteristics for embodied conversational agents in health. Results highlighted key avenues for future research, including the fact that more research is needed on all design characteristics to advance the field. Notably, the review by ter Stal et al [15] included studies using any research design and studies where participants viewed stimuli but did not necessarily interact with a virtual assistant.

### Objectives

The evidence base for the use of interactive virtual health assistants is rapidly growing in both size and quality. In particular, experimental research designs with interactive virtual assistants are being reported increasingly, which should provide clearer evidence of the influence of design characteristics on user experience. A scoping review methodology offers an explicit, systematic means to overview this large and diverse body of literature using rigorous methods to minimize bias [16]. In this study, we seek to undertake the first scoping review of design characteristics of virtual health assistants, with a view to bring together the strongest evidence available regarding the effects of design characteristics on the user experience of interactive virtual health assistants. In particular, the aims of our scoping review are as follows:

1. Provide an overview of all the experimental research examining how design characteristics of virtual health assistants affect user experience
2. Summarize research findings of experimental research examining how design characteristics of virtual health assistants affect user experience
3. Identify whether research supports making recommendations for the design of virtual health assistants

Bringing together the available evidence on how design characteristics affect the user experience of virtual health assistants will assist researchers and software developers in making decisions about the look and feel of their software and developing the most user-friendly and effective virtual health assistants.

## Methods

This review is reported according to the PRISMA-ScR (Preferred Reporting Items for Systematic Reviews and Meta-Analyses Extension for Scoping Reviews) checklist [17].

### Eligibility Criteria

Eligibility criteria were designed using the population, intervention, comparator, and outcome framework (population: adults; intervention: virtual health assistant; comparator: design characteristics; and outcome: user experience) [18]. Original research articles in peer-reviewed journals and full-length conference papers were included.

#### Population

Studies with adult samples (aged ≥18 years) were included.

#### Intervention

Studies examining virtual health assistants were included. For this review, we considered virtual health assistants to be any virtual assistant aimed at the health consumer (general population or patient) relating to the prevention, management, or treatment of any physical or mental health condition, as well as clinical research. Virtual health assistants were included if they functioned on any electronic device (eg, smartphone, computer, and headset). *Wizard of Oz* virtual assistants (where the user believes they are interacting with a computer-automated virtual assistant, but the virtual assistant is operated by a human [19]) were included.

#### Comparator

Studies comparing design characteristics between ≥2 versions of a virtual health assistant were included. For this review, we defined design characteristics as characteristics of the virtual assistant that influence its *look and feel* without affecting its core content, purpose, or function. Examples of design characteristics include visual cues such as whether the virtual health assistant has an avatar (ie, an image that represents the virtual assistant), language style, and interaction modality (ie, text or speech). Between- and within-subject experimental designs were included.

#### Outcome

Studies evaluating user experience outcomes were included. For this review, we defined user experience to include self-reported evaluations of the virtual assistant or the user’s interaction with the virtual assistant that indicated a more positive or negative experience (eg, trustworthiness, likeability, enjoyment, and ease of use), affect, intentions to continue using the virtual assistant, and objective measures of user engagement (eg, frequency, duration, or nature of the interaction with the virtual health assistant). Only quantitative data were included.

### Exclusion Criteria

Dissertations, review articles, conference abstracts, and studies with children were excluded. Virtual assistants used for training or educating medical professionals, as well as robots with a physical body, were excluded. Studies were excluded if participants did not interact with the virtual health assistant; that is, they did not provide any input into the system. Studies were also excluded if the virtual health assistant was not the main component of the health program. Studies were excluded if they evaluated only 1 version of a virtual assistant (ie, nonexperimental research design with no comparator) or if they compared a virtual assistant to a human. Dependent variables that were not associated with a more positive or negative user experience—for example, those used as *manipulation checks* (eg, where participants were asked to confirm whether a realistic-looking assistant was indeed more realistic looking than a cartoon-style assistant)—were excluded.

### Information Sources and Search Strategy

A cross-disciplinary search of the literature was conducted on June 4, 2020, and included 5 electronic databases across the fields of health and information technology: Web of Science, MEDLINE, Embase, PsycINFO, and ACM Digital Library. Search terms for virtual assistant AND design characteristics were included in the search strategy (Table 1). Eligibility specifying the virtual assistant related to *health*, user experience outcomes, and experimental study design was assessed at screening. Searches were limited to the English language with no limit on publication date. Reference lists of the included studies and other key papers in the field were searched to identify further studies (pearling).

**Table 1:** Search terms.

Search category	Search terms
Virtual assistant	“conversational agent*” OR “conversational system*” OR “dialog system*” OR “dialogue system*” OR “assistance technolog*” OR “relational agent*” OR “virtual agent*” OR “virtual assistant*” OR “embodied agent*” OR chatbot*
Design characteristics	anthropomorphi* OR *humanness* OR *personality* OR emotion* OR *empathy* OR *sympathy* OR *humour* OR *humor* OR *language* OR linguistic* OR *communication* OR *“conversational tone”* OR *voice* OR *speech* OR *avatar* OR *“profile picture”* OR *face* OR *facial* OR graphic* OR *appearance* OR *“visual design”* OR *animation* OR *interface* OR button* OR menu* OR emoji* OR emoticon* OR *“human factors”*

### Evidence Selection and Data Charting

Search results from each database were imported into EndNote (Clarivate) [20], in which duplicates were removed. Studies were screened based on title and abstract. Studies that met the eligibility criteria progressed to full-text screening. The full texts of the studies were then screened to determine final eligibility. Articles were screened by 1 of 2 raters. Raters screened a randomly generated selection of 20 articles in duplicate, and the agreement was 100%. A custom form was developed and used for data charting (Multimedia Appendix 1). Extracted data included population, sample size, age, gender, study country, cultural background, health domain, purpose of the virtual assistant, name of the virtual assistant, *Wizard of Oz* design, device used, animated character, output modality, input modality, whether the interaction was scripted (whether participants were told what to say), duration of interaction, experimental design, and study results. If articles included multiple studies, data extraction was completed only for studies meeting the eligibility criteria. Where multiple eligible studies were included in an article, data were extracted separately. Where relevant outcomes were measured but not compared statistically between experimental conditions, authors were contacted to provide additional information.

### Data Synthesis

Study characteristics were compiled for all the studies included in the review. Where a study was reported in multiple articles, articles were compiled as 1 study with a primary reference indicated, as well as an indication of additional references. To facilitate data synthesis across diverse research designs, overarching categories were constructed to describe the health domains, design characteristics, and outcomes. Retrospective thematic analysis was used to identify similar health domains, design characteristics, and outcomes to construct the relevant categories. After data extraction was completed, lists of all reported health domains, design characteristics, and outcomes were compiled. After familiarization with the data, the first author sorted them into similar categories using an inductive approach (ie, directed by the data with no preconceived categories). These categories were reviewed with the senior author, refined, and named.

Data were synthesized descriptively. Descriptive statistics were used to summarize the body of research. A matrix of the design characteristics and outcome categories was constructed to summarize the research findings. Results in the matrix were based on statistical results reported in the articles. Where interactions were examined (eg, in factorial designs or examining interactions with participant characteristics), main effects were included in the matrix. Studies could report results for 1 or multiple outcomes within a particular outcome category. Results were categorized as positive, negative, or no effect. Where studies reported multiple results in a single outcome category, they were categorized as positive if all multiple outcomes showed positive effects, mixed positive if multiple outcomes were reported with both positive and nonsignificant effects, negative if all multiple outcomes showed negative effects, mixed negative if multiple outcomes were reported with both negative and nonsignificant effects, and no effect if multiple outcomes showed no significant effects.

Authors from 2 studies provided additional data on measures that were not compared between experimental groups. Independent sample *t* tests (2-tailed) were conducted, and the results were included in the matrix. In total, 4 studies did not present a statistical analysis comparing relevant experimental conditions; therefore, these studies are included only in the text description.

## Results

### Overview

**Figure 1:**
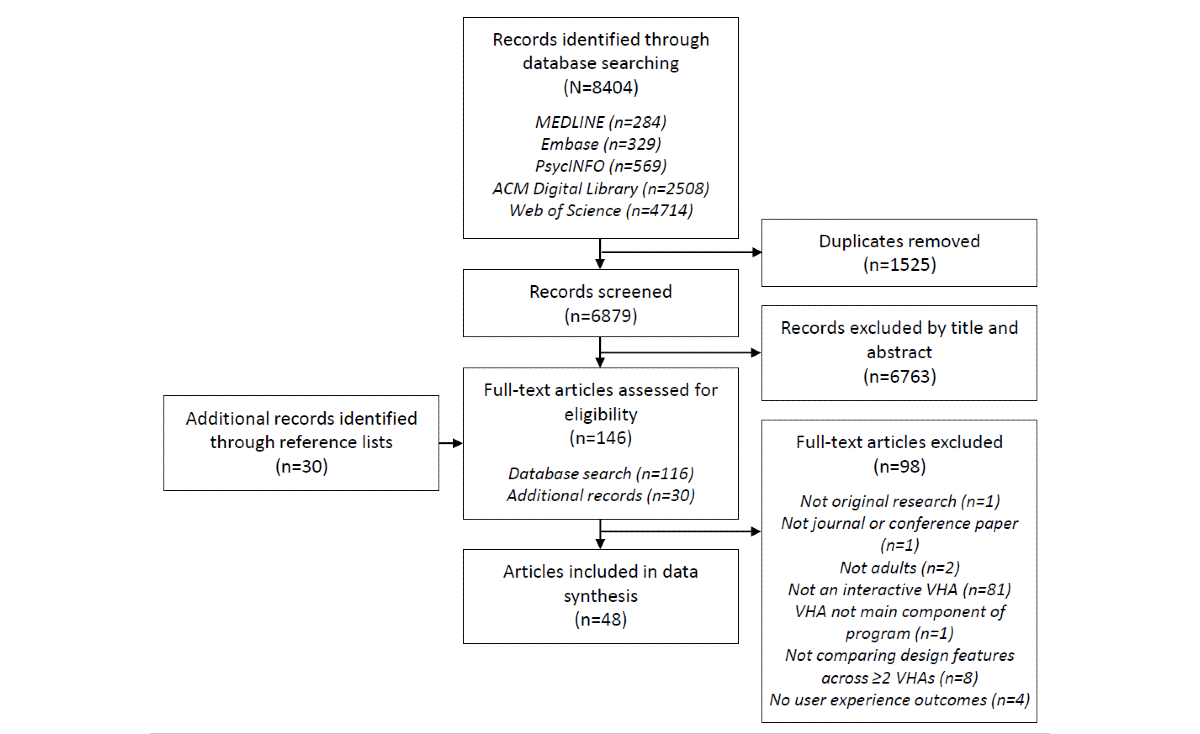
PRISMA (Preferred Reporting Items for Systematic Reviews and Meta-Analyses) flowchart. VHA: virtual health assistant.

The search identified 6879 articles after duplicates were removed. Of the 6879 articles, 6763 (98.31%) were deemed ineligible based on title and abstract screening. We identified 30 additional records through reference lists. In total, 146 articles (116/6879, 1.69% from the database search plus 30 from reference lists) were screened at full text. Of the 146 articles, 98 (67.1%) were deemed ineligible; 81 (55.5%) did not examine an interactive virtual health assistant, 8 (5.5%) did not compare design features between ≥2 virtual health assistants, 4 (2.7%) did not report user experience outcomes, 2 (1.4%) were not adult samples, 1 (0.7%) did not report original research, 1 (0.7%) was not a journal of conference paper, and 1 (0.7%) did not have the virtual health assistant as a main component of the program. Of the 146 articles, a final 48 (32.9%) articles were included in the scoping review (Figure 1). From the 48 articles, 45 unique studies were identified (5 studies were reported in multiple articles, whereas 3 articles contained multiple studies).

Multimedia Appendix 2 [21-68] provides an overview of the participant characteristics and study designs for all studies included in the review. Table 2 summarizes the characteristics of the body of research. The virtual assistants used in the research were categorized into 8 health domains: physical activity (aimed to increase exercise), nutrition (aimed to improve diet), alcohol consumption (aimed to reduce alcohol consumption), mental health (eg, aimed to improve mood), medical information or treatment (eg, discussed colorectal cancer screening), sexual health (eg, provided advice about sexually transmitted infections), multiple health behaviors (eg, aimed to improve both exercise and diet), and other (eg, aimed to prevent carpal tunnel). A total of 27 design characteristics were examined in the literature. These were categorized into 5 categories: visual design (eg, realism, age, and body shape of an animated avatar), interface design (eg, input modality), conversational style and relational behavior (eg, empathy and relational behavior and personality), combined visual and conversational design (eg, variability in language and background scene assessed simultaneously), and cultural and organizational affiliation (eg, culturally tailored argumentation and appearance; see Table 3 for the full list of design characteristics). We identified 140 outcome variables, which were categorized into nine categories: virtual assistant personality traits (eg, credible and intelligent), relationship (eg, intimacy and relationship closeness), ease of use (eg, cognitive load and ease of use), satisfaction (eg, enjoyment, satisfaction, and usefulness), emotion (eg, positive and negative affect), use intention (eg, intention to keep using the virtual assistant), engagement (eg, interaction duration), and disclosure (eg, self-disclosure detail and intimacy; see Multimedia Appendix 3 for a full list of outcomes by category). Outcome assessment most frequently used Likert scales, with server logs and conversation transcripts used to assess engagement and disclosure.

Most studies were conducted in the United States, with a greater number of studies conducted during more recent years (ie, between 2017 and 2020). Several authors led multiple studies (ie, Bickmore [25-27,29,31], Creed [34,35], Olaffsson, [53,54], Ring [56-58], and Zhou [67,68]). Most studies examined conversational style and relational behavior or visual design and assessed outcomes in the categories of personality, satisfaction, relationship, and use intention. Virtual assistants most frequently related to mental health and physical activity. Those addressing multiple health behaviors frequently examined physical health and nutrition together. Most virtual assistants had an animated avatar and used speech output and multiple-choice input. Most virtual assistants were automated (did not use a *Wizard* *of* *Oz* design), and participant input was not scripted. Studies were most frequently conducted with between 21 and 100 participants in a single session using a between-subjects design, where participants were allocated to evaluate 1 version of the virtual assistant. Participants were most frequently from the general population, with a larger proportion of females than males. Studies were most often published in conference proceedings in fields related to interdisciplinary research on intelligent virtual agents and human–computer interactions, with fewer published in health-related fields.

**Table 2:** Summary of study characteristics (N=45).

Study Characteristics				Value, n (%)
**Year**				
	2017-2020		18 (40)	
	2013-2016		11 (24)	
	2009-2012		10 (22)	
	2005-2008		6 (13)	
**Country**				
	United States		25 (56)	
	United Kingdom		4 (9)	
	Other		7 (16)	
	Not available		9 (20)	
**Sample size**				
	1-20		6 (13)	
	21-50		16 (36)	
	51-100		14 (31)	
	101-200		5 (11)	
	201-500		4 (9)	
**Duration**				
	Single session		37 (82)	
	Multiple sessions		8 (18)	
**Health domain**				
	Mental health		11 (24)	
	Physical activity		10 (22)	
	Multiple health behaviors		7 (16)	
	Medical information or treatment		6 (13)	
	Nutrition		4 (9)	
	Sexual health		2 (4)	
	Alcohol consumption		1 (2)	
	Other		4 (9)	
**Design category^a^**				
	Conversational style and relational behavior		22 (49)	
	Visual design		12 (27)	
	Interface design		6 (13)	
	Cultural and organizational affiliation		5 (11)	
	Combined visual and conversational design		2 (4)	
**Outcome category**				
	Personality		30 (67)	
	Satisfaction		20 (44)	
	Relationship		19 (42)	
	Use intention		17 (38)	
	Engagement		11 (24)	
	Ease of use		8 (18)	
	Emotion		7 (16)	
	Disclosure		5 (11)	
**Virtual assistant characteristics**				
	**Animated avatar**			
		Yes^b^	31 (69)	
		No	14 (31)	
	**Output**			
		Speech^c^	33 (73)	
		Text	11 (24)	
		Not available	1 (2)	
	**Input**			
		Multiple choice	26 (58)	
		Speech^c^	11 (24)	
		Text	7 (16)	
		Not available	1 (2)	
	**Wizard of Oz**			
		No	37 (82)	
		Yes	8 (18)	
	**Scripted**			
		No	41 (91)	
		Yes	4 (9)	

^a^N sums to >45 studies and 100% because 2 studies examined design characteristics in multiple categories.

^b^Includes studies where at least one experimental condition used an animated avatar.

^c^Includes studies where at least one experimental condition used speech.

**Table 3:** Summary of research findings (N=41).

Design characteristics		Values, n (%)	Outcomes (effect)								
			Personality	Relationship	Ease of use	Satisfaction	Emotion	Use intention	Engagement	Disclosure	
**Visual design**											
	Animated avatar (vs no visual representation)	3 (7)	Mixed negative [48]^a^,[62] No significant effect [51]	Mixed negative [48]^a^,[62]	No significant effect [48]^a^	No significant effect [48]^a^,[51,62]	No significant effect [48]^a^,[51]	No significant effect [48]^a^	—^b^		—
	Realistic (vs cartoon)	4 (10)	Positive [64] Mixed negative [57] No significant effect [57,62]	No significant effect [62]	No significant effect [64]	Mixed positive [64] No significant effect [62]	—	Positive [64] No significant effect [57]^c^ (2 studies)	—		—
	Human (vs robot)	1 (2)	Mixed negative [62]	Mixed negative [62]	—	Mixed negative [62]	—	—	—		—
	Younger (vs older)	1 (2)	No significant effect [64]	—	No significant effect [64]	Mixed positive [64]	—	No significant effect [64]	—		—
	Fat (vs slim)	1 (2)	Positive [63]	No significant effect [63]	—	—	—	No significant effect [63]	—		—
	Familiar (vs unfamiliar)	1 (2)	Mixed negative [64]	—	No significant effect [64]	Mixed negative [64]	—	No significant effect [64]	—		—
	Medical professional attire (vs casual)	1 (2)	Positive [55]	Positive [55]	—	—	—	Positive [55]	—		—
	Medical office (vs empty room)	1 (2)	Mixed positive [55]	No significant effect [55]	—	—	—	No significant effect [55]	—		—
	Variability in camera angle (vs no variability)	3 (7)	Mixed positive [58] No significant effect [58]^c^ (2 studies)	—	—	—	—	—	No significant effect [58]^c^ (3 studies)		—
**Interface design**											
	Speech input (vs text or multiple choice)	3 (7)	No significant effect [33]	No significant effect [33]	Mixed negative [32]	—	—	—	Positive [52]		—
	Motion initiated (vs user initiated)	1 (2)	—	Positive [56]	—	—	Positive [56]	—	No significant effect [56]		—
	Polite notification ringtone (vs impolite)	1 (2)	Positive [25]	—	—	—	—	Positive [25]	—		—
**Conversational style and relational behavior**											
	Empathy and relational behavior (vs none)	7 (17)	Positive [48,51] Mixed positive [31] No significant effect [40]^d^,[49]	Mixed positive [48] No significant effect [27,49]	No significant effect [48]	Positive [48] Mixed positive [27,31,51] No significant effect [49]	Mixed positive [27,39] No significant effect [48,51]	Positive [31,48] No significant effect [27]	No significant effect [40]		Positive [31]
	Emotional expression (vs none)	3 (7)	Positive [34,43]	No significant effect [34,35,43]	—	No significant effect [43]	No significant effect [34]	—	—		—
	Self-disclosure (vs none)	3 (7)	Positive [47]^e^ No significant effect [29]	Positive [44,47]	—	Positive [47] Mixed positive [29]	—	—	Positive [29,47]		Mixed positive [47]^e^ Positive [44]
	Personality (various)^f^	3 (7)	Positive [61] No significant effect [36]	Mixed positive [60] No significant effect [36]	Positive [61] No significant effect [36]	No significant effect [36]	No significant effect [36]	No significant effect [36,60]	No significant effect [36]		—
	Conversation memory (vs none)	2 (5)	Mixed positive [36] No significant effect [23]	Mixed positive [36]	No significant effect [36]	No significant effect [23,36]	No significant effect [36]	No significant effect [23,36]	No significant effect [23,36]		—
	Humor (vs none)	1 (2)	Mixed positive [36]	No significant effect [36]	No significant effect [36]	Mixed positive [36]	No significant effect [36]	No significant effect [36]	No significant effect [36]		—
	Emojis (vs none)	1 (2)	No significant effect [37]	—	—	No significant effect [37]	—	—	No significant effect [37]		—
	Rap (vs none)	1 (2)	Mixed negative [53]	Mixed positive [53]	—	No significant effect [53]	—	No significant effect [53]	—		—
	Participant control of facial and vocal expression (vs none)	1 (2)	—	—	No significant effect [26]	Mixed positive [26]	—	—	—		—
	Constrained to positive user response options (vs negative responses allowed)	1 (2)	No significant effect [54]	No significant effect [54]	—	Mixed negative [54]	—	No significant effect [54]	—		—
**Combined visual and conversational design**											
	Personification (name, static avatar, and conversational language vs none)	1 (2)	—	—	—	—	—	—	—		Mixed negative [59]
	Variability in dialog structure, language, and scene (vs no variability)	1 (2)	Positive [29]	—	—	—	—	Positive [29]	Positive [29]		—
**Cultural and organizational affiliation**											
	Culturally tailored argumentation (vs not)	2 (5)	No significant effect [65]	—	—	Positive [50]	—	—	—		—
	Culturally tailored appearance (vs not)	3 (7)	No significant effect [65,67]	Negative [67]	No significant effect [67]	No significant effect [50,67]	—	No significant effect [67]	—		—
	Culturally tailored argumentation and scene combined (vs not)	1 (2)	No significant effect [68]	No significant effect [68]	No significant effect [68]	No significant effect [68]	—	No significant effect [68]	—		—
	Patient assistant (vs researcher or government employee)	1 (2)	Mixed positive [66]	—	—	Mixed positive [66]	—	Positive [66]	—		—

^a^Results indicated for nonempathetic avatar only (empathetic avatar had additional dialog to the no avatar condition).

^b^No study examined the combination of design characteristic and outcome.

^c^Multiple studies were reported in the article with similar results.

^d^Similar results were additionally reported at a different time point in the study [39].

^e^Similar results were additionally reported at a different time point in the study [46].

^f^Indicates any effects of personality (no consistent comparator).

Table 3 summarizes research findings grouped according to the design characteristic examined and the categories of outcomes measured. Where identical outcomes of a study were reported in multiple articles, the primary reference listed in Multimedia Appendix 2 was used. Additional references were used for outcomes that were not reported in the primary study. In total, 4 studies did not present a statistical analysis comparing the relevant experimental conditions; therefore, these studies are not included in Table 3.

The following paragraphs highlight key results from the studies presented in Table 3 and include a narrative synthesis of studies that were not presented in Table 3.

### Visual Design

Approximately 7% (3/41) of studies examined whether user experience differed using a virtual assistant with an animated avatar compared with using a text- or speech-only virtual assistant with no visual representation [48,51,62]. Findings were generally nonsignificant [48,51,62], with some mixed negative effects of using an animated avatar [48,62]. An additional study not included in Table 3 concluded that virtual assistants with an animated avatar were preferred over voice-only assistants; however, the analyses included both real and virtual assistants [45].

Approximately 22% (9/41) of studies examined the appearance of the animated avatar, and 10% (4/41) of studies examined whether user experience differed using a virtual assistant with a more realistic human avatar compared with a more cartoon human avatar [57,62,64]. Although some positive and mixed positive effects of using a more realistic avatar were found [64], more effects were nonsignificant [57,62,64], and 1 was negative [57]. The species of the avatar was examined by 2% (1/41) of studies, which found mixed negative effects of using a human avatar compared with using a robot avatar [62]. Age was examined by 2% (1/41) of studies, which found mixed positive effects of using an avatar with a younger appearance compared with using one with an older appearance on satisfaction but no significant effects on other outcomes [64]. Body shape was examined by 2% (1/41) of studies, which found a positive effect of a fat avatar compared with a slim avatar on personality traits but nonsignificant effects on other outcomes [63]. The familiarity of the avatar was examined by 2% (1/41) of studies, which found mixed negative and nonsignificant effects of using an avatar that looked like a health coach that participants met at the beginning of the session compared with using an unfamiliar avatar [64]. The avatar’s attire was examined by 2% (1/41) of studies, which found consistently positive effects of medical professional attire compared with casual attire [55].

The background scene behind the avatar was examined by 2% (1/41) of studies, which found mixed positive effects of representing a medical office compared with representing an empty room on personality but no significant effects on other measured outcomes [55]. Approximately 7% (3/41) of studies (all reported in 1 paper) examined whether variability in the *camera* position, distance, and focus was associated with user experience and found mostly nonsignificant effects [58].

### Interface Design

Approximately 7% (3/41) of studies examined the effects of input modality—whether the user communicates using speech, text, or multiple choice—on user experience and found a combination of positive, mixed negative, and nonsignificant effects of speech input compared with other modalities [32,33,52]. A menu-based virtual assistant was examined by 1 further study not included in Table 3, and it concluded that there were no differences in usability between speech and phone key press user input [42].

How the conversation between the virtual assistant and user was initiated was examined by 2% (1/41) of studies, which found positive and nonsignificant effects of automated motion initiation compared with user initiation [56]. The type of ringtone used to initiate a conversation with the user was examined by 2% (1/41) of studies, which found positive effects of more polite tones compared with less polite tones [25].

### Conversational Style and Relational Behavior

Approximately 17% (7/41) of studies examined empathy and relational behavior—empathetic verbal feedback and nonverbal behavior such as facial expressions and gestures [27,31,39,40,48,49,51]. Although some effects were nonsignificant [27,40,48,49,51], more effects were positive or mixed positive, with 71% (5/7) of studies showing at least some positive effect [27,31,39,48,51]. Approximately 7% (3/41) of studies examined emotional expression—the use of facial expression and voice to express emotion—and found some mixed positive effects [34,43] but more nonsignificant effects [34,35,43]. Approximately 7% (3/41) of studies examined self-disclosure—whether the virtual assistant tells the user information about themselves—and found mostly positive effects [29,44,47]. Approximately 7% (3/41) of studies examined personality [36,60,61]. Although some positive and mixed positive effects were found [60,61], most effects were nonsignificant [36,60].

Approximately 5% (2/41) of studies examined conversation memory—whether the virtual assistant remembered information from earlier conversation—and found some mixed positive effects [36] but mostly nonsignificant effects [23,36]. An additional study not included in Table 3 compared users’ first interactions when the virtual assistant did not recall their previous session and when the virtual assistant did recall their previous session [38]. The authors concluded that users were more positive when the virtual assistant recalled their session; however, the conversations were less personal.

Humor was examined by 2% (1/41) of studies, which found mostly nonsignificant effects of including humor compared with not including humor [36]. Using emojis was examined by 2% (1/41) of studies, which found no significant effects of using emojis compared with not using emojis [37]. Rap was examined by 2% (1/41) of studies, which found a combination of mixed positive, mixed negative, and nonsignificant effects of including rap compared with not including rap [53]. Allowing participants to control the virtual assistant’s facial and vocal expression was examined by 2% (1/41) of studies, which found nonsignificant and mixed positive effects compared with not allowing such control [26]. Approximately 2% (1/41) of studies examined constraining users to respond only positively to questions about their confidence and motivation compared with also presenting negative multiple-choice response options [54]. It found a combination of mixed negative and neutral effects of constraining users to positive responses. A further study not included in Table 3 examined whether user evaluations were more positive for a virtual assistant that changed behavior based on the user’s eye contact compared with a virtual assistant that always appeared attentive or always bored or that changed behavior randomly [41]. The authors concluded that changing based on the user’s eye contact seemed more normal than changing behavior randomly but did not confirm the hypothesis that changing behavior is more normal than unchanging behavior.

### Combined Visual and Conversational Design

Personification—the use of a name, static avatar, and conversational language—was examined by 2% (1/41) of studies, which found negative effects of personification on users’ disclosure [59]. Variability in dialog structure (the order of the conversation and the utterances used) and background scene was examined by 2% (1/41) of studies, which found consistently positive effects of variability compared with no variability [29].

### Organizational and Cultural Affiliation

Approximately 10% (4/41) of studies examined cultural tailoring—matching the culture of the virtual assistant to that of the user [50,65,67,68]. Approximately 5% (2/41) of studies examined cultural tailoring of the virtual assistant’s argumentation (eg, discussed culturally relevant topics) [50,65], and 50% (1/2) of those found a positive effect [50]. Approximately 7% (3/41) of studies examined cultural tailoring of the virtual assistant’s appearance and the household setting and found predominantly nonsignificant effects [50,65,67]. Culturally tailored background scene and argumentation combined were examined by 2% (1/41) of studies, which found no significant effects [68]. The organizational affiliation of the virtual assistant—who the virtual assistant claimed to be and the context provided in the background scene—was examined by 2% (1/41) of studies, which found positive effects of the virtual assistant being a patient assistant compared with the virtual assistant being either a member of the medical team conducting the research or a government employee [66].

## Discussion

### Principal Findings

This study aimed to provide an overview of experimental research examining how design characteristics of virtual health assistants affect user experience. This is a growing area of scientific endeavor with studies, taken together, examining highly diverse health domains, design characteristics, and outcomes. The most common health domains were physical activity and mental health, with relatively few virtual assistants related to specific health conditions. Approximately half of the studies were categorized as examining the design of conversational style and relational behavior, with the most common design characteristic researched being empathy and relational behavior. The most commonly measured outcomes were in the categories of personality traits, satisfaction, relationship, and use intention.

This study also aimed to summarize the research findings of experimental research examining how design characteristics of virtual health assistants affect user experience. Generally, research has been piecemeal, with few design characteristics having a sufficient body of evidence to draw conclusions about their effects on user experience. The 2 design characteristics that defy this are virtual assistants’ empathy and relational behavior and self-disclosure, which have been the focus of a good number of studies. Research suggests that all 3 (ie, empathy, relational behavior, and self-disclosure) are related to more positive user experience. Other design characteristics with emerging levels of evidence are having a more realistic human representation for an avatar and having medical attire for the avatar, both of which may potentially be related to more positive user experience. Finally, evidence to date suggests that using an animated avatar (compared with no avatar) and cultural tailoring may not affect user experience; however, more research is needed to explore these findings.

One of the clearest findings of this study was that the use of empathy and relational behavior in virtual health assistants appears to have positive effects on user experience. Empathy may help to build trust and rapport with the virtual assistant. The finding that empathy was associated with user satisfaction is in line with research indicating a positive association between empathy in real health care providers and patient satisfaction [69,70]. Results were not consistently positive; however, this may be related to differences between the virtual assistants. For example, for the outcome category personality traits, of the 5 studies examining empathy and relational behavior, 3 (60%) studies showing positive effects used animated avatars, including nonverbal relational behaviors [31,48,51]. In contrast, 40% (2/5) of studies showing no effects were text-only assistants [40,49]. It may be that users do not expect text-only assistants to show empathy; therefore, the presence or absence of empathy has no impact on the ratings of the virtual assistant. Alternatively, the effects of empathy may be diminished when nonverbal relational behaviors such as expression and gestures are not present.

Research suggests that virtual health assistants that use self-disclosure (ie, provide information about themselves) elicit a more positive user experience. Results were similar whether the autobiographical information was framed as being about the virtual assistant’s experience as a computer agent [44] or included human experiences that could not actually be true [29,47]. Self-disclosure is important for the formation of relationships [71], although research suggests that self-disclosure by a real counselor can have either positive or detrimental effects on a client’s perceptions of the counselor [72]. The finding that users respond positively to the autobiographical stories of a virtual health assistant supports the *computers are social actors* paradigm, where users display social responses to computers, although they know they are not human [73,74].

Research examining the realism of the animated avatar showed some positive effects; however, more were nonsignificant. The *uncanny valley* theory suggests that robots that appear almost but not quite human may elicit a negative emotional response and be less likable than those that are clearly nonhuman [75]. However, in this review, the study that used a photo-realistic representation in the realistic experimental condition [64] showed positive effects. More research is needed to examine how the realism of the avatar affects the user experience of virtual health assistants.

Results from 1 study suggest that dressing the avatar in medical attire results in a more positive user experience [55]. Although more research is needed to confirm this finding, this was a large study (n=308) with consistent results across all outcomes measures. Interestingly, the background setting for the avatar (medical office or empty room) had a mixed positive effect on only 1 out of 3 outcomes categories [55].

Research suggests that including an animated avatar has no effect or, in some cases, a negative effect on user experience. However, upon closer inspection, this may be because of the nature of the avatars used in the research and may also be affected by interactions between the animation and other virtual assistant characteristics. For example, Lisetti et al [48] showed that an animated avatar with a neutral facial expression and no empathetic dialog led to poorer user experience than a text-only virtual assistant, whereas an expressive and empathetic virtual assistant led to a better user experience than the text-only virtual assistant. Nguyen and Masthoff [51] reported similar findings; a nonempathetic animated virtual assistant and a nonempathetic text-only virtual assistant led to a similar user experience; however, an empathetic animated virtual assistant led to better user experience than an empathetic text-only virtual assistant. Taken together, it appears that users may expect a virtual assistant with a human-like representation to have empathy and human-like relational behaviors and have a poorer user experience when this expectation is not met.

Overall, the research did not show cultural tailoring to improve the user experience of virtual health assistants. Notably, although 75% (3/4) of studies included participants who were born overseas (in China [68], India [50], or a Spanish-speaking Latin-American country [65]), participants in all the studies lived in the United States. This may suggest that cultural tailoring is not required for different cultures living in the United States who have had exposure to Anglo-American culture, although more research could confirm this finding. Additional research is also needed to determine whether cultural tailoring affects user experience in other cultural contexts.

### Strengths and Limitations

This scoping review is the most rigorous attempt at synthesizing the literature regarding the effects of design characteristics on the user experience of virtual health assistants. It followed the PRISMA-ScR guidelines for scoping reviews and searched a large number of databases. It examined a broad range of design characteristics using the highest level of evidence—experimental research using only interactive virtual health assistants where participants were able to input into the system. However, we acknowledge that the use of specific search terms to capture virtual assistants and design characteristics could have omitted some results. It is also possible that other literary sources may have been available in other databases. In addition, qualitative data were excluded. This enabled a structured approach to synthesizing the data based on statistical significance but may have omitted some important views on user experience.

Although the breadth of the review is a major strength, the heterogeneity of the included studies makes it difficult to synthesize and interpret the results. There was considerable heterogeneity in the purpose of the virtual assistants studied. Optimal design techniques may differ among different health domains. For example, although no overall effect of using emojis was found, the difference in ratings of confidence between using text-only and text with emojis depended on whether the virtual assistant was discussing physical or mental well-being [37]. In addition, some health conditions were not represented in the studies, for example, neurocognitive impairments such as dementia. There was also significant heterogeneity in the outcomes measured. The most commonly measured outcomes were in the categories of personality, satisfaction, relationship, and use intention. Few studies examined the ease of use, engagement, or disclosure. Although interface design may play a key role in determining the ease of use, other design characteristics such as the visual appearance of an avatar may not be expected to affect the ease of use. More research examining how users interact with the virtual assistant (engagement and disclosure), particularly using objective measures, may complement subjective ratings of the virtual assistant and interaction.

An additional limitation of the literature is that some studies combined a set of similar characteristics into 1 condition, making it difficult to ascertain which characteristic might be responsible for the effects on user experience. For example, research on empathy and relational behavior frequently included verbal empathy with nonverbal relational behaviors. In addition, in most studies, participants evaluated the virtual assistant after interacting during a single session. Programs that aim to promote health behavior change or provide support for a health condition are often designed for ongoing use. Additional research should examine how design characteristics affect user experience over time. Most virtual assistants had animated avatars and speech output; however, over half constrained user input to selecting from predefined response options. Constraining user input requires simpler programming and removes the risk of errors occurring when the virtual assistant misinterprets the user’s input or cannot formulate a response to a query that is outside the bounds of its programmed knowledge [76]. Natural language processing enables users to communicate using unconstrained text or speech and enables more natural user-directed communication. Virtual assistants using natural language processing have been commonly used in health care [77] and, with rapid advancements in artificial intelligence, are likely to become increasingly sophisticated. More research should examine the design and user experience of these types of virtual health assistants.

### Recommendations

Research demonstrates that design characteristics affect the user experience of virtual health assistants; therefore, researchers and software developers should carefully consider the look and feel of a virtual health assistant during development and testing. On the basis of the results of this scoping review, the following recommendations for designing virtual health assistants and advancing the field of research may be useful for health researchers and software developers:

1. Design virtual health assistants to express verbal empathy, for example, understanding of the user’s feelings
2. Design virtual health assistants to disclose personal information about themselves to the user, for example, information about their past and personal preferences
3. Consider designing a human avatar to be more realistic with medical professional attire
4. If designing an animated virtual health assistant, it should display nonverbal relational behaviors, for example, emotional facial expressions, gestures, and mutual gaze
5. If empathy and relational behaviors are unable to be incorporated, consider that an animated avatar may not be beneficial or cost-effective
6. Engage in formative research with the target audience and adopt a user-centered design approach to ensure that the software meets the needs and preferences of the user
7. Conduct further systematic research to replicate and extend previous findings, particularly with longitudinal research designs with repeated user interactions, objective engagement outcomes, and virtual assistants with natural language processing capabilities

### Conclusions

Virtual health assistants can provide health information and support on demand and may be applied in the future to a wide variety of purposes such as providing public health information, health education, supporting patients with chronic health conditions, and assisting with healthy lifestyle behavior change. This scoping review examined experimental research assessing how design characteristics of virtual health assistants affect user experience. This is a rapidly growing field of research but is difficult to synthesize and interpret because of the heterogeneity of studies. Nonetheless, certain design characteristics have emerged as important for improving user experience. Preliminary recommendations suggest that programming virtual health assistants to show empathy, display nonverbal relational behaviors, and disclose personal information about themselves may result in a more positive user experience. The decision to include an animated avatar should consider whether the avatar can display empathy and nonverbal relational behaviors. Future research is required to improve our understanding of the relationship between design characteristics and user experience of virtual health assistants, particularly with longitudinal research designs with repeated user interactions.

## Appendix

Multimedia Appendix 1 Data charting form.

Multimedia Appendix 2 Characteristics of studies included in the scoping review.

Multimedia Appendix 3 Outcome categories.

## Abbreviations

PRISMA-ScR: Preferred Reporting Items for Systematic Reviews and Meta-Analyses Extension for Scoping Reviews
