# Supplementary material for: Improving User Experience of Virtual Health Assistants: Scoping Review
Source: J Med Internet Res. 2021 Dec 21;23(12):e31737. doi: 10.2196/31737 (PMC8734926; doi:10.2196/31737)
Supplement: Multimedia Appendix 2 [file jmir_v23i12e31737_app2.docx]

Multimedia Appendix 2. Characteristics of studies included in the scoping review.

| **Reference** | **Description of program** | **Description of sample** | **Experimental method** | **Description of virtual assistant** | **Outcomes and results** |
| --- | --- | --- | --- | --- | --- |
| Bellur & Sundar 2017 [23]. Journal article. | Multiple health behaviours: Conducts a health risk assessment and provides feedback on health behaviour (diet, exercise, drug, alcohol, HIV- and AIDS-related risk factors). Single session. | Sample size: total n=172. Population: University students. Age: M=20.5, SD=1.19. Gender: 17% male. Country: USA. Cultural background: 71% Caucasian, 6% African American, 5% Hispanic, 16% Asian, 2% other. | Conversational stye and relational behaviour: Conversation memory.  3 × 2 between-person factorial design:  Interactivity:  1) Low: Does not display user input.  2) Medium: Displays most recent user input.  3) High: Displays full conversation on screen. Refers to conversation e.g., “Previously, you mentioned…”  Turn-taking:  1) Turn-taking: Used verbal turn-taking cues e.g., “OK, let’s talk about exercise”.  2) No turn-taking: No turn-taking cues. | Name NA, automated, device NA, no image or animated avatar, text output, multiple choice input, not scripted. | = Appealing, exciting, content quality, content value, fun, enjoyment, immersion, control. |
| Bickmore, Mauer et al. 2007 [25]. Conference paper. | Other: Aims to prevent carpel tunnel by reminding users to rest their wrists from a computer task. Single session. | Sample size: total n=29; Population: general. Age: range=18-30. Gender: 48% male. Country: USA. Cultural background: NA. | Interface design: Polite initiation tone.  Within-person; 4 conditions:  Four alert sounds used to signal the start of the conversation:  AUDIO1: Very polite (a subtle “ping”) to AUDIO4: Very impolite (a loud klaxon). | Name NA, automated, pocket PC, unrealistic animated human, text output, multiple choice input, not scripted. | + Polite, annoying, desire to continue using. |
| Bickmore & Picard 2005 [31]. Journal article.  *Also reported in Bickmore, Gruber et al. 2005 journal article [24]; Bickmore & Picard 2004 conference paper [30].* | Physical activity: Aims to increase exercise through education, goal setting, self-monitoring, and problem solving. Daily for 30 days. | Sample size: total n=91; relational agent n=33; non-relational agent n=27; control group n=31. Population: adults interested in becoming physically active. Age: M=24.8, SD=7.4. Gender: 45% male. Country: USA. Cultural background: NA. | Conversational stye and relational behaviour: Empathy and relational behaviour.  Between-person; 3 conditions:  1) Relational agent: Verbal relational behaviours (social dialogue, empathetic feedback, meta-relational communication, humour, close forms of address) and nonverbal immediacy behaviours (visually closer proximity, more frequent facial animation, gesture, head nods, and gaze at user).  2) Non-relational agent: No verbal and minimal non-verbal relational behaviours.  3) Control group: No access to the agent. | Laura, automated, computer, stylised unrealistic animated human, speech output, multiple choice input, not scripted. | + Liking, working alliance-bond, relationship closeness, usage intention, sent farewell, sentimental farewell.  = Working alliance-composite, working alliance-task, working alliance-goal, usefulness. |
| Bickmore & Ring 2010 [26]. Conference paper. | Other: Assists users to author health behaviour change stories for preconception counselling. Single session. | Sample size: total n=9. Population: African American females. Age: range=20-24. Gender: 0% male. Country: USA. Cultural Background: 100% African American. | Conversational stye and relational behaviour: User control of facial and vocal expression.  Within-person; 5 conditions:  1) Control text condition: Story is entered and displayed as text with no virtual assistant.  2) Agent: Story is told by the virtual assistant with automatically generated prosody and non-verbal behaviour with neutral facial expression.  3) Face: User controls facial expression.  4) Voice: User controls prosody (speed, volume, baseline pitch, emphasis, pause).  5) All: User controls expression and prosody. | Gladys, automated, computer, unrealistic animated human, speech output, text input, scripted. | + Helpful, satisfied.  = Easy, confidence, express self. |
| Bickmore & Schulman 2007 [27]. Conference paper. | Mental health: Aims to improve mood following a negative mood induction by discussing the user’s mood. Single session. | Sample size: total n=15. Population: general. Age: range=18-30. Gender: 46% male. Country: USA. Cultural background: NA. | Conversational stye and relational behaviour: Empathy.  Within-person; 2 conditions:  1) Empathetic agent: Provides empathic feedback to mood rating, including happy facial display for ‘great’ and concerned facial display for other states.  2) Non -empathetic expressive agent: Asks users for a free speech input. Neutral responses and facial display. | Louise, Wizard-of-Oz, screen, unrealistic animated human, speech output, speech input, not scripted. | + Satisfaction, positive affect.  = Cared for by virtual assistant, liking, desire to continue using, negative affect, heart rate, skin conductivity. |
| Bickmore, Schulman et al. 2010 [29]. Journal article.  Study 1. | Physical activity: Aims to increase walking through exercise counselling. Daily for up to 4 years (M=102 days before and 126 days after cross-over). | Sample Size: total n=24; variable n=10; non-variable n=14. Population: older adults. Age: 55-75. Gender: 29% male. Country: USA. Cultural background: NA. | Combined visual and conversational design: Variability in behaviour and background scene.  Randomised cross-over design; 2 conditions:  1) Variable: Dialogue structures, utterance forms and background scenes are randomly selected from multiple options for each interaction.  2) Non-variable: Same dialogue structure, utterances, and background scene for each interaction. | Karen, automated, computer, unrealistic animated human, output NA, multiple choice input, not scripted. | + Number of conversations, desire to continue using, repetitiveness. |
| Bickmore, Schulman et al. 2010 [29].  Study 2.  *Also reported in Bickmore et al. 2009 conference paper [28].* | Physical activity: Aims to increase walking through exercise counselling. Daily (M=29 days). | Sample size: total n=26; first-person n=13; third-person n=13. Population: older adults. Age: range=54-67. Gender: 19% male. Country: USA. Cultural background: 80% Caucasian, 20% African American. | Conversational stye and relational behaviour: Self-disclosure.  Between-person; 2 conditions:  1) First-person (self-disclosure): Virtual assistant presents a narrative as their own life story.  2) Third-person: Virtual assistant presents a narrative as stories about a friend. | Karen, automated, computer, unrealistic animated human, speech output, multiple choice input, not scripted. | + Enjoyment, completed conversations.  = Dishonest, look forward to using. |
| Chen et al. 2020 [32]. Journal article. | Multiple health behaviours: Collects information on physical activity and diet that users calculate from hypothetical scenarios. Single session. | Sample size: total n=50. Population: university staff and students. Age: range=18-64. Gender: 46% male. Country: Australia. Cultural background: NA. | Interface design: Speech input.  2 x 2 within-person factorial design:  Interaction modality:  1) Keyboard and mouse input.  2) Speech input.  Task complexity:  1) Simple problem solving: Few items for arithmetic; shown in same window.  2) Complex problem solving: More items for arithmetic; shown in different window. | Name NA, automated, static clipart image, text output, text or speech input (depending on condition), not scripted. | - Cognitive load, system usability (overall, usability, learnability), task completion time.  = User error rate. |
| Cho 2019 [33]. Conference paper. | Sexual health: Provides answers to health questions. Single session. | Sample size: total n=53; smart home device with voice n=18; smartphone with voice n=18; smartphone with text n=17. Population: university students. Age: range=19-23, M=20.2, SD=0.9. Gender: 34% male. Country: USA. Cultural background: NA. | Interface design: Speech input.  3 x 2 factorial design:  Interaction modality (between-person):  1) Smart home device with voice.  2) Smartphone with voice.  3) Smartphone with text.  Information sensitivity (within-person):  1) Low sensitivity: Questions related to allergies, cold, and flu.  2) High sensitivity: Sexual health questions. | Google Assistant, automated, smartphone or smart home device, no image or animated avatar, text or speech output and input (depending on condition), scripted questions. | = Perceived social presence, attitudes towards the voice assistant. |
| Creed & Beale 2012 [34]. Journal article. | Nutrition: Aims to improve diet through advice and goal setting. Single session. | Sample size: total n=50; emotion n=25; no emotion n=25. Population: university students. Age: NA. Gender: 40% male Country: UK. Cultural background: NA. | Conversational stye and relational behaviour: Emotional expression.  Between-person; 2 conditions:  1) Emotion: Uses facial expression and voice (speech rate, pitch) to express happiness, warmth, and concern, and neutral emotions.  2) No emotion: Neutral facial expression and voice. | Rachael, automated, device NA, realistic animated human, speech output, multiple choice input, not scripted. | + Caring likeability.  = Trustworthiness, intelligence, positive affect, felt supported. |
| Creed et al. 2015 [35]. Journal article. | Nutrition: Aims to improve fruit and vegetable consumption through advice, goal setting, and problem solving. Includes educational resources and self-monitoring tools. Weekly for 7 weeks. | Sample size: total n=44; emotion n=20; no emotion n=24. Population: university students. Age: M=30.5, SD=9.3. Country: n=41 UK and n=3 other. Cultural background: NA. | Conversational stye and relational behaviour: Emotional expression.  Between-person; 2 conditions:  1) Emotion: Uses facial expression and voice (speech rate, pitch average, pitch range) to express happiness, warmth, and concern, and neutral emotions.  2) No emotion: Neutral facial expression and voice. | Rachael, automated, device NA, realistic animated human, speech output, multiple choice input, not scripted. | = Working alliance-composite, working alliance-bond, working alliance-task, working alliance-goal. |
| De Boni et al. 2008 [36]. Journal article. | Physical activity: Aims to increase exercise by helping the user identify and overcome barriers to exercise. 5 sessions over 2 weeks. | Sample size: total n=374; relationship maintenance on n=189; relationship maintenance off n=185. Population: internet savvy adults seeking to increase physical activity. Age: range=18-56. Gender: 43% male. Country: UK. Cultural background: NA. | Conversational stye and relational behaviour: Conversation memory, humour, personality.  2 x 2 x 2 factorial design:  Conversation memory (between-person):  1) Conversation memory on: Refers to user by name, referred to previous conversations.  2) Conversation memory off: None of the above.  Humour (within-person):  1) Humour: Uses jokes and self-depreciation.  2) No humour: No jokes or self-depreciation.  Personality (within-person):  1) Dominant: Shorter, blunt sentences and strong words.  2) Submissive: Longer, more gentle sentences and softer words.  Analyses examine personality matching:  1) Matched to user’s personality.  2) Not matched to user’s personality. | Solution Advisor, automated, computer, no image or animated avatar, text output, text input, not scripted. | Outcomes: polite, supportive, reliable, impersonal, abrupt, incompetent, appropriate humour, professional, sincere, informative, patient, reassuring, uncaring, trustworthy, honest, considerate, genuine, dependable, adaptive, dominant, submissive, approachable, persuasive, motivating, long-winded, irrelevant, intrusive, recognition, uncomfortable, similar to me, easy to learn, not easy to understand, easy to follow, required concentration, easy to engage, frustrating to use, control, satisfied, helpful, enjoyable, unsettled, better than expected, desire to use again, prefer human.  Conversation memory:  + Polite, professional, considerate, genuine, approachable, recognition.  = All else.  Humour:  + Appropriate humour, too impersonal, too abrupt, enjoyable.  = All else.  Personality:  = All. |
| Fadhil et al. 2018 [37]. Conference paper. | Multiple health behaviours: Collects information on the user’s physical and mental wellness. Single session. | Sample size: total n=58; group A n=29; group B n=29. Population: general. Age: range=18-60, M=29.9, SD=11.9. Gender: 48% male. Country: NA. Cultural background: NA. | Conversational stye and relational behaviour: Emojis.  Between-person; 2 conditions:  1) Group A: Mental wellbeing dialogue with emojis and physical wellbeing dialogue in plain text.  2) Group B: Mental wellbeing dialogue in plain text and physical wellbeing dialogue with emojis. | CoachAI, automated, computer, static clipart image, text output, multiple choice input, not scripted. | = Enjoyment, attitude,  confidence, interaction time. |
| Ferland & Koutstaal 2020 [38]. Conference paper. | Mental health: Discusses the user’s schedule, projected (morning session) and retrospective (afternoon session) stress. 4 sessions over 2 days. | Sample size: total n=11. Population: young adults. Age: M=18.5. Gender: 27% male. Country: USA. Cultural background: 27% Caucasian, 9% Asian, 18% African American, 9% multi-racial. | Conversational stye and relational behaviour: Conversation memory.  Within-person; 2 conditions:  1) Unadapted model: Day 1: does not remember the morning session in the afternoon. Day 2: remembers the previous day and morning session.  2) Adapted model: Both days: remembers the sessions. | Name NA, Wizard-of-Oz, computer, embodiment NA, speech output, input NA, not scripted. | Results not classified. Word count, emotional tone, authenticity, clout, analytical thinking. |
| Ghandeharioun et al. 2019 [40]. Conference paper.  *Also reported in Ghandeharioun et al. 2019 conference paper [39].* | Mental health: Assesses the user’s mood and provides activities for mental wellness. 5 times per day for 3 weeks. | Sample size: total n=39; emotion aware n=19; control n=20. Population: general. Age: range=16-49, M=29.4, SD=7.9. Gender: 82% male. Country: USA. Cultural background: NA. | Conversational stye and relational behaviour: Empathy and relational behaviour.  Between-person: 2 conditions:  1) Emotion-Aware: Provides an empathetic, emotionally-expressive response to user mood, including emojis.  2) Control: Provides a neutral response with no emotion or emojis. | Emma, automated, mobile phone, no image or animated avatar, text output, multiple choice input, not scripted. | + Momentary positive emotion.  = Positive and negative affect, response latency, response frequency, overall perception. |
| Grillon & Thalmann 2008 [41]. Conference paper. | Mental health: Conducts a practice conversation for social phobia. Single session. | Sample size: total n=12. Population: general. Age: range=25-35. Gender: NA. Country: NA. Cultural background: NA. | Conversational stye and relational behaviour: Variability.  Within-person, 4 conditions:  1) Attentive: Always attentive and demonstrates a positive attitude.  2) Bored: Always looks away and seems bored.  3) Random: Randomly changes attitude (attentive versus bored).  4) Tracked: Changes attitude depending on user eye-contact (attentive when looked at, bored when not looked at). | Name NA, automated, projector screen, realistic animated human, speech output, speech input, not scripted. | Results not classified. Interested, engaged, friendly, normal. |
| Grover et al. 2009 [42]. Conference paper. | Medical information or treatment: Provides health information to caregivers of children with HIV. Single session. | Sample size: total n=33. Population: caregivers of children with HIV. Age: range=22-61, M=34. Gender: 3% male. Country: Botswana. Cultural background: NA. | Interface design: Speech input.  Within-person; 2 conditions:  1) Automatic speech recognition user input.  2) Key press user input. | Open Phone, Wizard-of-Oz, telephone, no image or animated avatar, speech output, multiple choice input (key press or spoken depending on condition), not scripted. | Results not classified. Response time, task completion rate, task completion time, turns to reach information, timeouts, repeats, barge-ins, use of exit menu, use of main menu. |
| Guadagno et al. 2011 [43]. Journal article. | Mental health: Discusses a personal topic. Single session. | Sample size: total n=38. Population: undergraduate women. Age: M=20.2, SD=1.6. Gender: 0% male. Country: USA. Cultural background: NA. | Conversational stye and relational behaviour: Emotional expression (smile).  2 x 2 between-person factorial design:  Smile:  1) Smile: Smiles when appropriate.  2) No smile: Never smiles.  Interaction partner:  1) Agent: Users told the counsellor is computer-generated.  2) Avatar: Users told the counsellor is a research assistant represented by a virtual human. | Beth, Wizard-of-Oz, head mounted display, realistic animated human, speech output, speech input, not scripted. | = Empathy, general positivity, supportiveness, likeability, comfort, copresence, trust, satisfaction, enjoyment, satisfaction with interaction modality. |
| Kang S-H & Gratch 2011 [44]. Conference paper. | Mental health: Virtual counsellor interview; asks the user questions requiring intimate self-disclosure. Single session. | Sample size: total n=57. Population: general. Age: M=30.7, SD=10.1. Gender: 47% male. Country: USA. Cultural background: NA. | Conversational stye and relational behaviour: Self-disclosure.  Between-person; 3 conditions:  1) High disclosure: Precedes all 10 questions with autobiographical backstory.  2) Low disclosure: Precedes 3 questions with low intimacy backstory.  3) No disclosure: Provides no backstory. | Name NA, Wizard-of-Oz, computer, realistic animated human, speech output, speech input, not scripted. | + Co-presence, social attraction, self-disclosure. |
| Kim et al. 2019 [45]. Conference paper. | Medical information or treatment: In-home patient care assistant (turns on/off devices, checks vital signs, discusses pain, food and physical activity). Single session. | Sample size: total n=32. Population: general. Age: range=19-41, M=25.1, SD=5.6. Gender: 78% male. Country: USA. Cultural background: NA. | Visual design: Animated avatar.  2 (between-person mediation) x 2 (within-person embodiment) factorial design; 4 conditions:  1) Virtual embodied: A virtual human with synthesized voice, gestures and facial expression superimposed in the environment.  2) Virtual voice: Synthesised voice with no display.  3) Real embodiment: A human care assistant physically present in the room.  4) Real voice: A human care assistant communicating remotely through voice only. | Name NA, Wizard-of-Oz, Microsoft Hololens and laptop, animated human (virtual embodied condition only), speech output, speech input, scripted. | Results not classified. Social richness attributes, usability, engagement, working alliance-bond, social presence, willingness to use for 18 activities. |
| Lee, Yamashita, Huang, Fu 2020 [47]. Conference paper.  *Also reported in Lee, Yamashita & Huang 2020 journal article [46].* | Mental health: Prompts journaling (moods, experiences, gratitude, stress, and anxiety) and asks sensitive mental health questions. Daily for 4 weeks. | Sample size: total n=47; no self-disclosure n=16; low self-disclosure n=15; high self-disclosure n=16. Population: university students. Age: range=20-27, M=23. Gender: 40% male. Country: NA. Cultural background: NA. | Conversational stye and relational behaviour: Self-disclosure.  Between-person; 3 conditions:  1) No self-disclosure: No small talk.  2) Low self-disclosure: Small talk with more general responses and less feeling/thought responses.  3) High self-disclosure: Small talk with a high level of self-disclosure including deeper feelings, thoughts, or information. | Name NA, automated, mobile phone or other chosen device, static handshaking image, text output, text input, not scripted. | + Trust, intimacy, enjoyment, word count in journaling and sensitive questions, self-disclosure in sensitive questions (feelings).  = Self-disclosure in journaling (thoughts, information, feelings) and sensitive questions (thoughts, information). |
| Lisetti et al. 2013 [48]. Journal article.  *Also reported in Amini et al. 2013 conference paper [22]; Amini et al. 2014 conference paper [21].* | Alcohol consumption: Aims to reduce alcohol consumption using motivational interviewing. Single session. | Sample size: total n=81; empathic counsellor n=26; non-empathic counsellor n=25; text only=30. Population: university students who had at least 1 alcoholic drink in the past month. Age: NA. Gender: 63% male. Country: USA. Cultural background: 55% White, 27% Hispanic, 16% African American, 2% Asian. | Visual design: Animated avatar. Conversational stye and relational behaviour: Empathy and relational behaviour.  Between-person; 3 conditions:  1) Empathic counsellor: Empathic verbal and non-verbal reactions (emotional facial expressions; head gesture; smile; head posture mimicry; eyebrow movement; mutual gaze; and lip synchronized verbal reflection).  2) Non-empathic counsellor: Neutral facial expression, no empathy.  3) Text-only: Text-only web page frames. | Amy, automated, computer, realistic animated human, speech or text output (depending on condition), multiple choice input, not scripted. | Animated avatar (vs non-empathetic):  - Perceived Intelligence, perceived sociability.  = Attitude, intention to use, perceived enjoyment, perceived ease of use, perceived usefulness, perceived safety, social presence, likability, trust, anxiety, social influence.  Empathy:  + Attitude, trust, likability, perceived intelligence, social presence, perceived sociability, perceived safety, perceived enjoyment, perceived usefulness, intention to use.  = Social influence, perceived ease of use, anxiety. |
| Liu & Sundar 2018 [49]. Journal article. | Sexual health: Provides advice on concerns about sexually transmitted infections. Single session. | Sample size: total n=88. Population: general. Age: range=18-52, M=25.8, SD=6.8 Gender: 27% male. Country: USA. Cultural background: NA. | Conversational stye and relational behaviour: Empathy and relational behaviour.  Between-person; 4 conditions:  1) Advice only: No perspective or compassion.  2) Sympathy: Feels bad or sorry for the user.  3) Cognitive empathy: Recognizes and acknowledges the user’s feelings and situation.  4) Affective empathy: Understands how and why the user feels a certain way. | Name NA, automated, computer, static robot image, text output, text input, scripted. | = perceived sadness, emotion recognition, sincerity, perceived understanding, spine tingling, eeriness, felt supported, likeability, intelligence, attitude, novelty, message supportiveness, message effectiveness. |
| Murali et al. 2020 [50]. Conference paper. | Physical activity: Aims to increase exercise. Single session. | Sample size: total n=40. Population: adults born in India who migrated to the USA. Age: M=25.2, SD=1.8. Gender: 60% male. Country: USA. Cultural background: 100% Indian migrants. | Cultural and organisational affiliation: Culturally tailored appearance and argumentation (Indian).  2 x 2 between-person factorial design:  Appearance:  1) Indian: Indian appearance.  2) American: Caucasian appearance.  Argumentation:  1) Indian: Indian accent, discusses collectivist values.  2) American: American accent, discusses individualist values. | Raveena/Angela, automated, touch screen computer, animated human, speech output, speech input, not scripted. | Appearance:  = satisfaction.  Argumentation:  + satisfaction. |
| Nguyen & Masthoff 2009 [51]. Conference paper. | Mental health: Aims to reduce negative mood after a mood induction exercise by discussing the exercise and the user’s mood. Single session. | Sample size: total n=84. Population: general. Age: M=25.4, SD=7.9. Gender: 44% male. Country: UK. Cultural background: NA. | Visual design: Animated avatar. Conversational stye and relational behaviour: Empathy.  2 x 3 between-person factorial design: Animated avatar:  1) Animated: Animated human.  2) Non-animated: No visual representation.  Empathy:  1) Empathy: Asks caring and polite questions. Comforts users by validating their feelings.  2) Empathy and expressivity: As per Empathy condition and encourages users to freely expand on their feelings.  3) Non-empathy: Ignores user’s feelings. Asks distracting questions. | Mary, automated, computer, realistic animated human (animated condition only), speech output, multiple choice input, not scripted. | Animated avatar:  = Caring, likeable, trustworthy, enjoyment,  liking of language, appearance, gestures, and voice, positive affect, negative affect.  Empathy:  + Caring, likeable, trustworthy, empathetic, enjoyment.  = Liking of language, appearance, gestures and voice, positive affect, negative affect. |
| Novielli et al 2010 [52]. Journal article. | Nutrition: Assesses and provides advice on user’s diet. Single session. | Sample size: total n=60; spoken n=30; written n=30. Population: university students. Age: range=21-28. Gender: NA. Country: Italy. Cultural background: NA. | Interface design: Speech input.  Between-person; 2 conditions:  1) Spoken: Spoken input method.  2) Written: Keyboard input method. | Valentina, Wizard-of-Oz, Laptop or touchscreen, realistic animated human, speech output, text or speech input (depending on condition), not scripted. | + number of moves, number of characters per move. |
| Olafsson et al 2017 [53]. Conference paper. | Multiple health behaviours: Aims to improve exercise and diet through health counselling. Single session. | Sample size: total n=83. Population: general. Age: range=18-25, M=22.8, SD=2.4. Gender: 60% male. Country: USA. Cultural background: 79% Asian, 18% white, 2% Hispanic, 1% black. | Conversational stye and relational behaviour: Rap.  Within-person; 4 conditions:  1) RE: Rap included, exercise dialogue.  2) RN: Rap included, nutrition dialogue.  3) NoRE; No rap, exercise dialogue.  4) NoRN: No rap, nutrition dialogue.  Randomized into first conversation (e.g. RE), then used opposite condition (e.g. NoRN). | Name NA, automated, device NA, realistic animated human, speech output, multiple choice input, not scripted. | + Relationship closeness.  - Trust.  = Knowledgeable, natural, similar, satisfaction, willingness to continue using, liking, choice of assistant for final conversation, number of turns during final conversation. |
| Olafsson et al 2019 [54]. Conference paper. | Multiple health behaviours: Aims to increase physical activity or fruit and vegetable consumption using motivational interviewing. Single session. | Sample size: total n=39. Population: general. Age: M=24.6, SD=1.9. Gender: 45% male. Country: USA. Cultural background: NA. | Conversational stye and relational behaviour: Constrained to positive response options.  Within-person, 2 conditions:  1) Coerced positive change talk: Restricts user to choose positive options when asked to express their confidence and motivation levels.  2) Non-coerced change talk: Positive and negative options are presented. | Katherine/Emily, automated, tablet, realistic animated human, speech output, multiple choice input, not scripted. | - Preference.  = Satisfied, desire to continue working, trust, liking, knowledgeable, natural, relationship closeness, similar. |
| Parmar et al. 2018 [55]. Conference paper. | Medical information or treatment: Discusses the importance of having a healthcare proxy. Single session. | Sample size: total n=308; casual/empty n=73; casual/full n=85; prof/empty n=80; prof/full n=70. Population: general. Age: range=18-76, M=38, SD=12.3. Gender: 43% male. Country: USA. Cultural background: NA. | Visual design: Attire and background scene.  2 x 2 between-person factorial design:  Attire:  1) Casual: Avatar dressed in casual clothing  2) Professional: Avatar dressed as a medical professional.  Setting:  1) Empty: Empty room.  2) Full: Doctor’s office. | Name NA, automated, computer, realistic animated human, speech output, multiple choice input, not scripted. | Professional attire:  + Professional, caring, credible, trustworthy, friendly, appropriate appearance, reassuring, comfortable talking to, felt like a face-to-face conversation, desire to continue using.  Doctors office:  + Professional, reassuring.  = Credible, caring, trustworthy, friendly, appropriate appearance, comfortable talking to, felt like a face-to-face conversation, desire to continue using. |
| Ring et al. 2015 [56]. Journal article. | Mental Health: Aims to provide in-home social support, encourage positive affect, and promote physical activity. As desired for 1 week. | Sample size: total n=14. Population: older adults living alone. Age: range=56-75, M=65. Gender: 21% male. Country: USA. Cultural background: NA. | Interface design: Motion initiation.  Between-person; 2 conditions:  1) Proactive: Motion sensor triggers conversation.  2) Passive: Users initiate the conversation. | Tanya, automated, touch screen computer, stylised animated human, speech output, multiple choice input, not scripted. | + Comfortable loneliness, happiness.  = Frequency of conversation, duration of conversation. |
| Ring et al. 2014 [57]. Conference paper.  Study 1. | Medical information or treatment: Discusses the importance of colorectal cancer screening. Single session. | Sample size: total n=67. Population: general. Age: NA. Gender: 54% male. Country: NA. Cultural background: NA. | Visual design: Realism.  2 x 2 within-person factorial design:  Shading:  1) Realistic: Realistically-shaded human avatar.  2) Cartoon: Cartoon-shaded human avatar.  Dialogue:  1) Medial dialogue.  2) Social dialogue. | Name NA, automated, device NA, realistic or cartoon animated human (depending on condition), speech output, multiple choice input, not scripted. | Medial dialogue:  - Friendliness.  = Familiarity trustworthiness, likability, caring, appropriateness, desire to continue working with. |
| Ring et al. 2014 [57].  Study 2. | Medical information or treatment: Discusses the importance of colorectal cancer screening. Single session. | Sample size: total n=47. Population: general. Age: NA. Gender: 66% male. Country: NA. Cultural background: NA. | Visual design: Realism.  2 x 2 within-person factorial design:  Proportions:  1) Realistic: Realistically-proportioned character.  2) Cartoon: Cartoon-proportioned character with large head and facial features.  Dialogue:  1) Medial dialogue.  2) Social dialogue. | Name NA, automated, device NA, unrealistic animated human, speech output, multiple choice input, not scripted. | Medial dialogue:  + Appropriateness.  - Friendliness.  = Familiarity trustworthiness, likability, caring, appropriateness, desire to continue working with. |
| Ring et al. 2016 [58]. Conference paper.  Study 1. | Physical activity: Aims to persuade the user to increase their exercise. Single session. | Sample size: total n=284; auto n=102; random n=88; static n=84. Population: general. Age: range 18-69, M=34.8. Gender: 58% male. Country: NA. Cultural background: NA. | Visual design: Variability in ‘camera’ angle.  Between-person; 3 conditions:  1) Auto: Camera location changes based on conversation topic.  2) Static: No camera motion.  3) Random: Random camera motion. | Name NA, automated, computer, realistic animated human, speech output, multiple choice input, not scripted. | + Attitudes  = Number of dialogue turns. |
| Ring et al. 2016 [58].  Study 2. | Physical activity: Aims to persuade the user to increase their exercise. Single session. | Sample size: total n=149; auto n=51; random n=44; static n=54. Population: general. Age: range 19-65, M=36.4. Gender: 56% male. Country: NA. Cultural background: NA. | Visual design: Variability in ‘camera’ angle.  Between-person; 3 conditions:  1) Auto: Camera location changes based on conversation topic. Camera distance changes based on emotional intensity of the conversation.  2) Static: No camera motion.  3) Random: Random camera motion. | Name NA, automated, computer, realistic animated human, speech output, multiple choice input, not scripted. | = Attitudes, number of dialogue turns. |
| Ring et al. 2016 [58].  Study 3. | Physical activity: Aims to persuade the user to increase their exercise. Single session. | Sample size: total n=99; auto n=37; random n=34; static n=28. Population: general. Age: range 19-74, M=38. Gender: 51% male. Country: NA. Cultural background: NA. | Visual design: Variability in ‘camera’ angle.  Between-person; 3 conditions:  1) Auto: Camera location changes based on conversation topic. Camera focus (depth of field) changed based on emotional intensity.  2) Static: No camera motion.  3) Random: Random camera motion. | Name NA, automated, computer, realistic animated human, speech output, multiple choice input, not scripted. | = Attitudes, number of dialogue turns. |
| Sannon et al. 2018 [59]. Conference paper. | Mental health: Asks the user about a recent stressor. Single session. | Sample size: total n=467. Population: general. Age: NA. Gender: NA. Country: NA. Cultural background: NA. | Combined visual and conversational design: Personification.  Between-person; 3 conditions:  1) Survey: Non-interactive static web form.  2) Non-personified chatbot: Chat window, no avatar image, no name.  3) Personified chatbot: Chat window, robot image, named “Taylor”, conversational language style. | Taylor (personified chatbot only), automated, device NA, static clipart image (personified chatbot only), text output, text input, not scripted. | - Disclosure detail.  = Disclosure intimacy. |
| Shamekhi et al. 2016 [60]. Conference paper. | Multiple health behaviours: Asks about daily tasks and provides advice to promote a healthy lifestyle. Single session. | Sample size: total n=102. Population: general. Age: range=19-66, M=33.2, SD=10.3. Gender: 70% male. Country: Canada and USA. Cultural background: NA. | Conversational stye and relational behaviour: Personality.  Within-person, 2 conditions:  1) High involvement: Talks quickly, short pauses, more verbose, more emotion, exaggerates, humour.  2) High considerateness: May pause before responding, speaks clearly and not too quickly, shows emotion without exaggeration.  Analyses examine personality matching:  1) Matched to user’s personality.  2) Not matched to user’s personality. | Name NA, automated, computer, no image or animated avatar, speech output, speech input, scripted. | + Engaging.  = Focussed, emotional, bored, trust, likeable, desire to continue. |
| Smestad et al 2019 [61]. Conference paper. | Nutrition: Aims to promote a healthy diet by assisting with meal planning. Single session. | Sample size: total n=16. Population: young couples living together. Age: range=25-40. Gender: 50% male. Country: Norway. Cultural background: NA. | Conversational stye and relational behaviour: Personality.  Between-person design; 2 conditions:  1) Chatbot A: Agreeable, extraverted personality.  2) Chatbot B: Low in agreeableness, extroversion, and neuroticism, moderate in openness and high in conscientiousness. | Bella, automated, device NA, static cartoon human image, text output, text input, not scripted. | + (chatbot A) pragmatic quality, hedonic quality-stimulation, hedonic quality-identity, attractiveness. |
| Strassmann et al. 2020 [62]. Journal article. | Multiple health behaviours: Assists in filling in a health diary (mood, physical activity, sleep, water intake, diet) and scheduling appointments. Single session. | Sample size: total n=130; 46 seniors; 86 students. Population: general. Age: seniors range=51-89, M=70.9, SD=9.1; students range=18-38, M=23.7, SD=3.8. Gender: 38% male Country: Germany. Cultural background: NA. | Visual design: Animated avatar, realism, species.  4 x 2 between-person factorial design:  Avatar appearance:  1) Voice only: No avatar.  2) Robot: Cartoon robot.  3) Cartoon human: Human with cartoon shading.  4) Realistic human: Human with realistic shading and higher resolution.  User age:  1) Students 2) Seniors | Billie/Character/Vince, Wizard-of-Oz, device NA, animated robot or cartoon human or realistic human (depending on condition), speech output, speech input, not scripted. | Outcomes: Competence, sociability, likeability, trustworthiness, perceived usefulness, attractiveness, liking, intention to use, trust, working alliance-bond, enjoyment, ease of use.  Animated avatar:  - Competence, sociability.  = All else.  Human:  - Likeability, sociability liking.  = All else.  Realistic:  = All. |
| van Vugt et al. 2006 [63]. Conference paper. | Other: Asks about the user’s knowledge on several health-related issues. Single session. | Sample size: total n=80; similar/ideal n=17; dissimilar/ideal n=14; similar/not ideal n=18; dissimilar/not ideal n=27. Population: university students. Age: M=23, SD=7.8. Gender: 30% male. Country: Netherlands. Cultural background: NA. | Visual design: Fat avatar.  2 x 2 between-person factorial design:  Valence:  1) Ideal: Thin avatar.  2) Not ideal: Fat avatar.  Similarity:  1) Similar: Avatar with same body shape as user.  2) Dissimilar: Avatar with different body shape to user. | René/Reneé, automated, computer, non-realistic human with four static poses but no facial expression, text output, text input, not scripted. | + Perceived ethics.  = User involvement, user distance, intentions to use. |
| van Wissen et al. 2016 [64]. Conference paper. | Medical information or treatment: Assists with chronic condition self-management (advice about physical activity, reminders for medication and appointments). Includes feature to track blood pressure and weight. Single session. | Sample size: total n=64. Population: older adults with a chronic health condition. Age: NA. Gender: 47% male. Country: Netherlands. Cultural background: 95% Caucasian. | Visual design: Realism, familiarity, and age.  3 x 2 factorial design:  Age/familiar (between-person):  1) Young unfamiliar: Image of a woman aged 30-35, not known to user.  2) Old unfamiliar: Image of a woman aged 55-60, not known to user.  3) Familiar: Image of a woman who explained the study in person.  Realism (within-person):  1) Realistic: Photograph.  2) Stylised: Stylised version of the photograph. | Name NA, automated, tablet, animated realistic or stylised human (depending on condition), speech output, multiple choice input, not scripted. | Outcomes:  Friendly, competent, attractive, trust, liking, satisfaction, easy to use, useful, useful health goal achievement, easier to manage health, useful for health management, like working with, makes life more interesting, help when necessary, continue interaction.  Younger age:  + Useful.  = All else.  Familiar:  - Friendly, useful, useful health goal achievement like working with.  = All else.  Realistic:  + Friendly, competent, attractive, trust, liking, satisfaction continue interaction.  = All else. |
| Yin et al. 2010 [65]. Conference paper. | Physical activity: Aims to increase exercise by discussing benefits and barriers to exercise. Single session. | Sample size: total n=43. Population: bi-lingual Anglo-American & Latino. Age: range=18-65. Gender: 56% male. Country: USA. Cultural background: 65% Latino, 35% Anglo-American. | Cultural and organisational affiliation: Culturally tailored appearance and argumentation.  2 x 2 between-person factorial design:  Language:  1) English.  2) Spanish.  Culture:  1) American: Anglo-American appearance and background image, born in New Hampshire, and discusses Northeastern University.  2) Latino: Latino appearance and background image and discusses family.  Analyses examine linguistic (preferred vs non-preferred language) and cultural congruity. | Katherine/Catalina, automated, device NA, realistic animated human, speech output, multiple choice input, not scripted. | Language:  = Trustworthiness.  Culture:  = Trustworthiness. |
| Zhang et al. 2017 [66]. Journal article. | Other: Explains patient consent documents for clinical research. Single session. | Sample size: total n=41. Population: general. Age: M=35.9, SD=16.7. Gender: 78% male. Country: USA. Cultural background: 59% Asian/Pacific Islander, 17% Black, 24% White. | Cultural and organisational affiliation: Organizational affiliation - Patient assistant:  Within-person; 3 conditions:  1) Patient assistant: Virtual assistant is a patient assistant; background shows another patient with virtual assistant.  2) Medical team: Virtual assistant is a member of the medical team providing the clinical trial; background shows a medical team.  3) Government employee: Virtual assistant is a Federal government employee who oversees the trial; background shows government building. | Name NA, automated, device NA, stylised animated human, speech output, multiple choice input, not scripted. | + Caring, trust (composite), liking, satisfaction with instructor, desire to continue.  = Trust (1 item), knowledgeability, appropriate amount of information, satisfaction with instructional experience, satisfaction with consent experience. |
| Zhou et al. 2014 [67]. Conference paper. | Medical information or treatment: Provides information for hospital discharge. Single session or daily while in hospital. | Sample size: total n=149; black agent n=67; white agent n=82. Population: hospital patients. Age: range=18-90, M=49. Gender: 49% male. Country: USA. Cultural Background: 54% Black, 27% White, 19% other. | Cultural and organisational affiliation: Culturally tailored appearance.  Between-person, 2 conditions:  Agent race:  1) Black agent: African American.  2) White agent: Caucasian appearance.  Analyses examine racial concordance:  1) Concordant: Both agent and user African American OR both not African American.  2) Non-concordant. | Elizabeth, automated, touchscreen computer, stylised animated human, speech output, multiple choice input, not scripted. | - Working alliance-bond.  = Satisfied, easy to talk to, liking, relationship closeness, trust, felt cared for, preference, similarity. |
| Zhou et al. 2017 [68]. Conference paper. | Physical activity. Aims to increase exercise by discussing benefits, barriers, and examples of exercise options. Single session. | Sample size: total n=49; American agent n=24; Chinese agent n=25. Population: Chinese adults living in the US. Age: range=19-34, M=26.1, SD=3.6. Gender: 47% male. Country: USA, Cultural Background: 100% born in China. | Cultural and organisational affiliation: Culturally tailored argumentation and background image combined (Chinese).  Between-person, 2 conditions:  1) American agent: American name, typical US living room, more hand gestures, culturally specific topics (e.g. local sport) and exercises (e.g. cycling).  2) Chinese agent: Chinese name, interior design with traditional Chinese elements, fewer hand gestures, culturally specific topics (e.g. Lunar New Year) and exercises (e.g. badminton). | Elsie/Meimei, automated, touchscreen computer, realistic animated human, speech output, multiple choice input, not scripted. | = Socially close, satisfied, desire to continue using, liking, easy talking to, interesting, relationship closeness, felt cared for, mutual understanding, motivating. |

Note: NA indicates Not available, + indicates positive effect, = indicates no effect, - indicates negative effect.
