# Supplementary material for: Improving User Experience of Virtual Health Assistants: Scoping Review
Source: J Med Internet Res. 2021 Dec 21;23(12):e31737. doi: 10.2196/31737 (PMC8734926; doi:10.2196/31737)
Supplement: Multimedia Appendix 3 [file jmir_v23i12e31737_app3.docx]

Multimedia Appendix 3. Outcome categories

| **Category** | **Outcomes** |  |  |
| --- | --- | --- | --- |
| **Virtual assistant personality traits** | Abrupt  Adaptive  Annoying  Appealing  Approachable  Appropriateness  Appropriate appearance  Appropriate humour  Appropriate information  Attitude  Attractive  Bored  Caring  Competent  Considerate  Content quality  Content value  Credible  Dependable | Dishonest  Dominant  Eeriness  Emotional  Empathy  Engaging  Ethics  Exciting  Focussed  Friendly  Genuine  Hedonic Quality  Honest  Impersonal  Incompetent  Informative  Intelligent  Intrusive  Irrelevant | Likeable  Overall perception  Patient  Uncaring  Long winded  Motivating  Natural  Polite  Positive traits  Professional  Reassuring  Reliable  Repetitiveness  Sadness  Sincere  Spine tingling  Submissive  Supportive  Trust |
|  |  |  |  |
| **Relationship** | Comfortable interaction  Copresence  Felt cared for  Felt supported  Intimacy  Perceived Safety Recognition | Relationship closeness  Similarity  Sociability  Social attraction  Social influence  Social presence | Socially close  Uncomfortable  Understanding  User distance  User involvement  Working alliance |
|  |  |  |  |
| **Satisfaction** | Better than expected  Confidence  Easier to manage health  Enjoyment  Express self  Fun  Helpful  Like working with  Liking | Liking of language  Liking of appearance  Liking of gestures  Liking of voice  Look forward to using  Makes life more interesting  Message effectiveness  Prefer human | Preference  Satisfaction with interaction  Satisfaction with virtual assistant  Useful  Useful goal achievement  Useful health management |
|  |  |  |  |
| **Emotion** | Anxiety  Heart Rate  Loneliness | Negative affect  Positive affect | Skin Conductivity  Unsettled |
|  |  |  |  |
| **Usage intention** | Desire to continue using | Intention to continue using |  |
|  |  |  |  |
| **Ease of use** | Cognitive load  Easy to engage  Easy to follow  Easy to learn  Easy to talk to | Easy to use  Frustrating to use  Not easy to understand Pragmatic Quality | Required concentration System usability  Task completion time  Task error rate |
|  |  |  |  |
| **Engagement** | Choice for additional conversation  Feeling in control Immersion  Interaction duration | No. characters per move No. completed conversations  No. moves | Response frequency  Response latency  Word count |
|  |  |  |  |
| **Disclosure** | Disclosure of stressful events  Self-disclosure detail  Self-disclosure intimacy | Self-disclosure feelings Self-disclosure level  Self-disclosure of information | Self-disclosure of thoughts  Sent farewell  Sentimental farewell |
